# Different antibiotic regimes in men diagnosed with lower urinary tract infection – a retrospective register-based study

**DOI:** 10.1080/02813432.2020.1794409

**Published:** 2020-07-20

**Authors:** Helena Kornfält Isberg, Katarina Hedin, Eva Melander, Sigvard Mölstad, Olof Cronberg, Sven Engström, Heidi Lindbäck, Thomas Neumark, Gunilla Stridh Ekman, Anders Beckman

**Affiliations:** a Department of Clinical Sciences, Family Medicine Malmö, Lund University, Malmö, Sweden; b Futurum, Region Jönköping County and Department of Health, Medicine and Caring Sciences, Linköping University, Linköping, Sweden; c Regional Center for Communicable Disease Control, Malmö, Sweden; d Department of Translational Medicine, Lund University, Malmö, Sweden; e Department of Research and Development, Region Kronoberg, Växjö, Sweden; f Department of Medical Sciences, Uppsala University, Uppsala, Sweden; g Regional Executive Officer's Staff – Coordination of Health Care, Kalmar, Sweden; h Strama Uppsala Region, Uppsala, Sweden

**Keywords:** Urinary tract infection, men, primary health care, therapy failure, recurrence, complication, antibiotic

## Abstract

**Objective:**

To compare the proportion of therapy failure, recurrence and complications within 30 days after consultation between men diagnosed with lower urinary tract infection (UTI) treated with narrow-spectrum antibiotics (nitrofurantoin or pivmecillinam) and broad-spectrum antibiotics (fluoroquinolones or trimethoprim or trimethoprim/sulfamethoxazole).

**Design:**

A retrospective cohort study based on data derived from electronic medical records between January 2012 and December 2015.

**Setting:**

Primary health care and hospital care in five different counties in Sweden.

**Patients:** A total of 16,555 men aged between 18 and 79 years diagnosed with lower UTI.

**Main outcome measures:**

Treatment with narrow-spectrum antibiotics was compared with broad-spectrum antibiotics regarding therapy failure, recurrence and complications within 30 days.

**Results:**

The median age of included men was 65 IQR (51–72) years. Narrow-spectrum antibiotics were prescribed in 8457 (40%) and broad-spectrum antibiotics in 12,667 (60%) cases, respectively. Therapy failure was registered in 192 (0.9%), recurrence in 1277 (6%) and complications in 121 (0.6%) cases. Therapy failure and recurrence were more common in patients treated with narrow-spectrum antibiotics and trimethoprim (*p* < 0.001), but no such difference could be detected regarding complications.

**Conclusion:**

There was no difference in incidence of complications within 30 days between men treated with narrow- or broad-spectrum antibiotics. Patients prescribed broad-spectrum antibiotics had lower odds of reconsultation because of therapy failure and recurrence. From current data, treatment with narrow-spectrum antibiotics seems to be an optimal choice regarding preventing complications when treating men with lower UTI.

**KEY POINTS**
- Complications such as pyelonephritis and sepsis are uncommon in men diagnosed with lower urinary tract infection treated with antibiotics.
- There was no difference in incidence of complications among men diagnosed with lower urinary tract infection treated with narrow- or broad-spectrum antibiotics.
- In spite of higher incidence of therapy failure and recurrence, treatment with narrow-spectrum antibiotics seems to be an optimal choice regarding preventing complications when treating men diagnosed with lower UTI.

## Introduction

The incidence of urinary tract infection (UTI) in men varies with age and is uncommon (0.9 to 2.4 cases per 1000 men per year) in those younger than 55 years [1,2]. UTI is more common in older men but still far less prevalent than among women [3,4]. Thus, studies on UTI in men are difficult to perform since it is problematic to find large enough populations in which to draw reliable conclusions. There is a lack of randomised controlled studies (RCTs) concerning proper antibiotic choice and duration of therapy in male UTI [5,6].

The most common bacteria causing UTI in men is *Escherichia coli* (*E. coli)*. During the past decades, the number of *E.coli* resistant to the former first-line antibiotics, fluoroquinolones, trimethoprim and trimethoprim/sulfamethoxazole (i.e. broad-spectrum antibiotics) have increased [5]. This has led to an increased risk of therapy failure and a need for new treatment guidelines for UTI among male patients. In 2017, there was a change in Swedish guidelines (preceded by a review in 2014) and treatment with pivmecillinam or nitrofurantoin (narrow-spectrum antibiotics) for seven days was recommended as first-line antibiotics in male lower UTI [5,7]. Fluoroquinolones should, according to the new guidelines, only be used in cases with febrile UTI and trimethoprim is no longer recommended as empirical treatment because of the resistance situation [5]. Guidelines from other countries, for example the UK (Nice Guidelines), still include trimethoprim as first choice antibiotic [8]. According to Swedish and international guidelines, urine cultures should always be performed in male UTI patients before antibiotics are taken [5,8].

The new treatment recommendations for male UTI are based largely on studies conducted in women and children, and on clinical data that support treatment with pivmecillinam and nitrofurantoin for 5–7 days in women with lower UTI [9]. The guidelines are based on limited evidence [5,7] with no existing study comparing narrow-, and broad-spectrum antibiotic treatment on clinical outcome in men with UTI.

The change in treatment recommendation has contributed to an alteration in treatment regime by prescribers. The sales of fluoroquinolones to men in 2017 decreased by 4.3% compared with 2016, while the sales of pivmecillinam and nitrofurantoin to men increased by 4.1% and 10.3%, respectively [10]. For patient safety reasons, it is important to evaluate the outcome in the treatment of lower UTI in men after the implementation of new treatment guidelines. As far as we know no studies have compared narrow-spectrum with broad-spectrum antibiotics in men with lower UTI.

Therefore, the aim of this study was to compare the proportion of therapy failure, recurrence and complications within 30 days after treatment for lower UTI between men treated with narrow-spectrum antibiotic and broad-spectrum antibiotics.

## Materials and methods

This retrospective study was based on information derived from Swedish electronic medical records (EMR). The population was 883,449 men in 2012 with a 2.9 percent increase until 2015. We included men from 289 primary healthcare centres and 20 hospitals in five counties in southern and central Sweden. Data for all men aged 18–79 years diagnosed with lower UTI or complications pertaining to UTI in primary health care (PHC) and hospital care from January 2012 to December 2015 were identified through the EMR databases of Jönköping, Kalmar, Kronoberg, and Uppsala county. From Skåne county, data on lower UTI diagnoses in primary care and complications diagnosed in primary care and hospital care were collected from December 2012 to December 2015. However, it was not possible to retrieve data on UTI index visits from hospital care from Skåne county. We extracted data on day of consultation or days of hospitalisation, diagnosis, antibiotic treatment, urine dip stick, C-reactive protein (CRP) and results from urine cultures. Microbiological analyses had been performed at five different laboratories (one in each county), all using the same approved conventional methods for the identification to the species level [11].

### Design

We identified eligible patients with a visit in PHC or hospital care, an ICD code indicating lower UTI (Supplementary material) and an adjacent antibiotic prescription. UTI diagnoses with an antibiotic prescription in the preceding 30 days were excluded. The same patient could be included in the study more than once.

**Figure 1.**
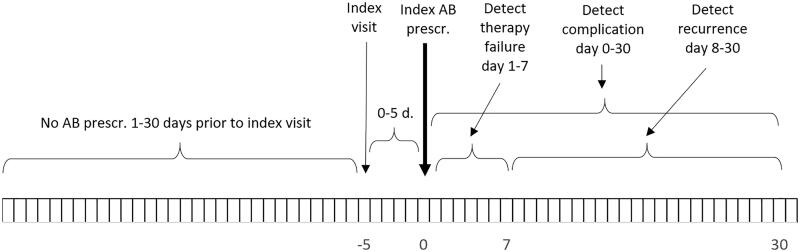
Observation time. To qualify as an index visit there should be no antibiotic prescriptions within the preceding 30 days. To qualify as an index antibiotic prescription, its date shall be within five days from the index visit (if a urine culture is to be analysed prior to antibiotic prescribing it might take up to five days from the index visit to the index antibiotic prescription) Therapy failure may be detected within day 1–7 from the index antibiotic prescription. Recurrence may be detected within day 8–30 from the index antibiotic prescription. A complication may be detected within 30 days from the index antibiotic prescription.

The study started on 1 January 2012 in four of the five included counties. In Skåne county, the study started on 8 December 2012. No visits during the first 30 days of the study were included, since there was no information on antibiotic prescribing for the preceding 30 days. Index visits were included until 31 December 2015. The follow-up period after an index UTI visit was for a maximum of 35 days after an index UTI visit (Figure 1).

### Definitions

**UTI diagnosis:** Patients who had a lower UTI diagnostic code recorded according to the International Classification of Disease and Related Health Problems-Tenth Revision (ICD-10) and KSH97-P (Classification of diseases and health problems) a short form of ICD-10 adopted to primary care in Sweden (Supplementary material).

**Index visits:** A visit to PHC or in hospital with a registered lower UTI diagnosis and a registered prescription of UTI relevant antibiotics on the day of visit or within five days.

**Antibiotic use:** UTI relevant antibiotics within five days from registered visit; the latest prescribed was used in the analysis. Prescribed antibiotics were registered according to the Anatomical Therapeutic Classification System (ATC) [12].

**Study antibiotic:** Antibiotics intended to be further compared in the study were divided into three different groups based on previous Swedish treatment guidelines for lower UTI in men concerning broad-spectrum antibiotics and treatment duration (Broad spectrum; trimethoprim <10 days or fluoroquinolones (ciprofloxacin) or trimethoprim/sulfamethoxazole ≥10 days) and the present treatment guidelines concerning narrow-spectrum antibiotics (Narrow spectrum; nitrofurantoin or pivmecillinam <10 days).

**Treatment duration:** Number of days of antibiotic treatment noted on the prescription.

**Therapy failure:** A new prescription of a different relevant UTI antibiotic within seven days from index antibiotic prescription and a new registered lower UTI diagnosis.

**Recurrence:** A new visit, a lower UTI diagnosis and an antibiotic prescription (the same day) 8 ≤ *d* ≤ 30 days from prior antibiotic prescription due to a UTI diagnosis.

**Complication:** A revisit in PHC or hospital care combined with a diagnosis of pyelonephritis or sepsis (Supplementary material) and an antibiotic prescription (the same day) within 30 days from the index antibiotic prescription.

**Microbiology:** Bacterial cultures and species determination were performed using approved conventional methods [11].

### Statistical analysis

The data collected in the study were analysed using Matlab 2019a (Mathworks, Natick, MA), Excel 2016 (Microsoft. Corp., Redmond, WA, USA), and SPSS Statistics 25 (IBM Corp. Released 2017. IBM SPSS Statistics for Windows, Version 25.0. Armonk, NY: IBM Corp).

Patient characteristics are described as median (IQR) for numerical variables and as numbers and per cent for categorical variables.

Comparisons between proportions of categorical variables in two independent groups were performed using the two-sided Chi-square test.

Binary logistic regression was used to model the correlation between therapy failure, recurrence and complications and antibiotic choice and other independent variables. Odds ratios (OR) with 95% CI were calculated.

Statistical significance was set at *p* < 0.05 (two-sided).

### Ethics

Confidentiality for patients was ensured by using an encrypted ID-number. The study was approved by the Regional Ethical Review Board in Lund, Sweden (Dnr 2016/462).

## Results

**Figure 2.**
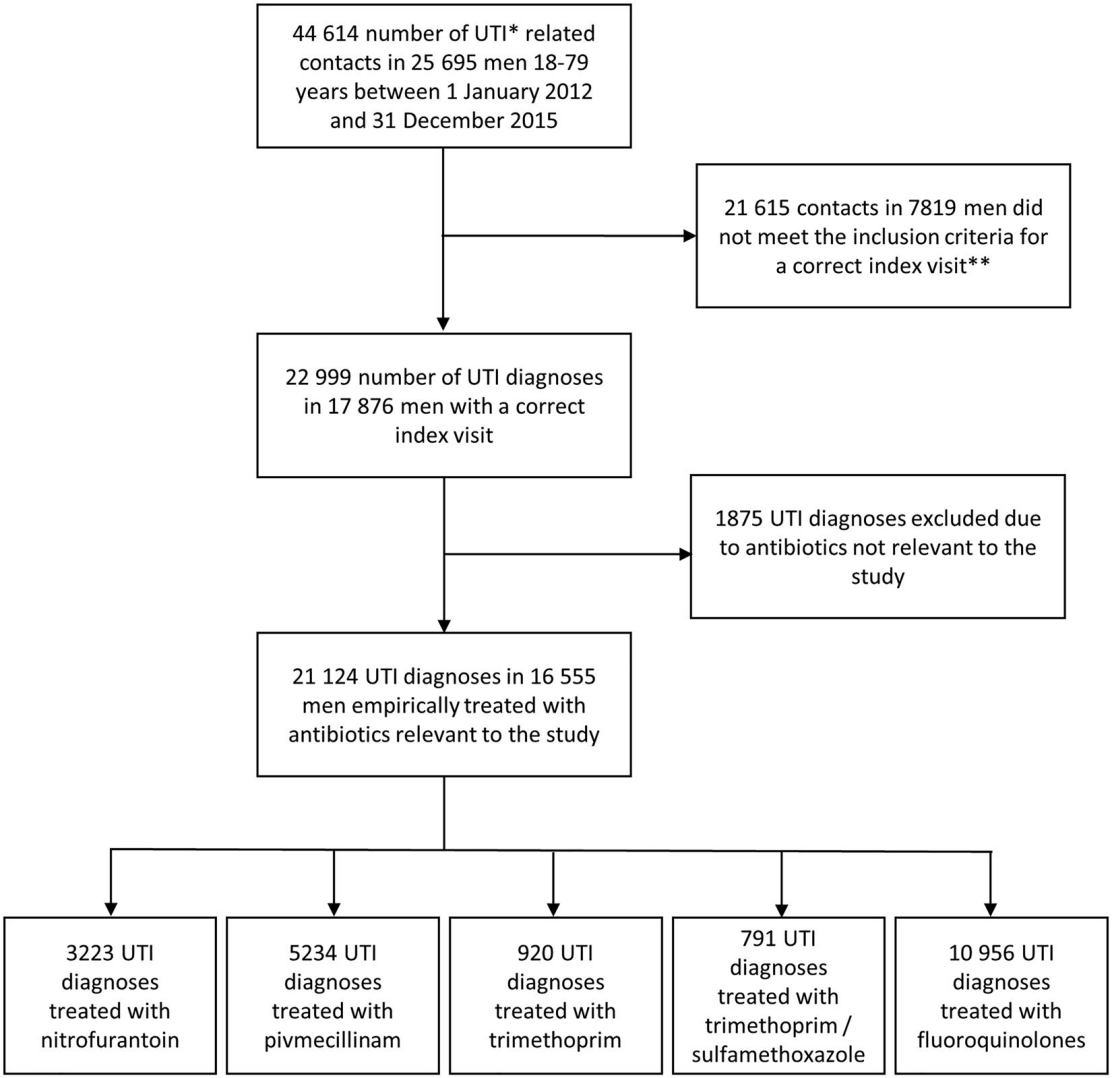
Flow chart of the inclusion process. *UTI: Urinary tract infection. **Inclusion criteria: Inclusion criteria were age 18–79 (<80) years, an index visit to primary health care or hospital combined with a defined diagnosis of lower UTI, and a prescription of UTI antibiotics on the day of visit or within five days from the consultation.

During the investigated years (2012–2015), a total of 44,614 visits diagnosed as lower UTI with a concurrent prescription of antibiotics among men 18–79 years were made in included counties (Jönköping, Kalmar Kronoberg, Skåne, Uppsala). Of these diagnoses, 22,999 met the study’s inclusion criteria for a correct index visit. In 21,124 of these index visits, antibiotics relevant to this study were prescribed. These visits were eligible for inclusion in the final study (Figure 2).

Most patients were handled in PHC (78%). The median age of included patients was 65.0 IQR (51–72) years (Table 1). Among the 16,555 patients observed during the four-year study period, a total of 13,711 (83%) patients had one single index visit, 2184 (13%) had two index visits and 497 (3%) three index visits due to lower UTIs.

**Table 1.** Description of the study population and outcome by prescribed antibiotic.

Antibiotic	Pivmecillinam	Nitrofurantoin	Trimethoprim	SXT^a^	Ciprofloxacin	Total
	*n* = 5234	*n* = 3223	*n* = 920	*n* = 791	*n* = 10956	*n* = 21 124
Age, years, median (IQR)	64 (49–72)	65 (49–72)	66 (53–73)	66 (56–72)	65 (53–72)	65 (51–72)
*Level of care, index*						
Primary health care *n* (%)	4815 (92.0)	3014 (93.5)	756 (82.2)	406 (51.3)	8188 (74.7)	17179 (81.3)
Hospital care *n* (%)	419 (8.0)	209 (6.5)	164 (17.8)	385 (48.7)	2768 (25.3)	3945 (18.7)
*Measurements*						
Median temperature^b^ Celsius (IQR)	36.9 (36.5–37.2)	36.9 (36.6–37.2)	36.8 (36.4–37.2)	37.5 (36.8–38.3)	37.0 (36.6–37.6)	37.2 (36.7–37.9)
CRP, mg/L^c^ median, (IQR)	7.0 (5.0–26.0)	7 (5–26.0)	11.0 (5.0–36.2)	41.0 (14.0–96.0)	40 (8.0–96.0)	24.0 (5–75)
Nitrite-positive^d^*n*(%)	796 (30)	400 (27)	111 (27)	130 (42)	1917 (39)	3354 (34)
Leukocytes posistive^e^*n*(%)	2310 (70)	1242 (67)	395 (76)	342 (78)	5124 (77)	9413 (74)
Urine culture analysed *n* (%)	2701 (52)	1613 (50)	512 (56)	503 (64)	6326 (58)	11 655 (55)
*Duration of therapy*						
0-9 days *n* (%)	4722 (90.4)	2839 (88.2)	677 (73.8)	357 (45.5)	5251 (48.0)	15073 (65.5)
10 days or more, *n* (%)	501 (9.6)	380 (11.8)	240 (26.2)	428 (54.1)	5679 (51.8)	7834 (34.1)
Median duration of therapy days (IQR)	7 (7–7)	5 (5–7)	7 (5–10)	10 (7–10)	10 (7–10)	7 (7–10)
*Outcome*						
Therapy failure *n* (%)	63 (1.2)	40 (1.2)	11 (1.2)	11 (1.2)	69 (0.6)	192 (0.9)
Recurrence *n* (%)	496 (9.5)	283 (8.8)	61 (6.6)	61 (6.6)	384 (3.5)	1277(6.0)
Complication *n* (%)	25 (0.5)	20 (0.6)	4 (0.4)	4 (0.4)	61 (0.6)	121 (0.6)

^a^ SXT: Trimethoprim/sulfamethoxazole.

^b^ Missing cases for temperature, 13,242 (62.7%) of all index patients.

^c^ CRP (C-reactive protein), missing cases 12,345 (58.4%).

^d^ Measured by dipstick test in urine, missing cases 11,118 (53%).

^e^ Measured by dipstick test in urine, missing cases 9413 (46%).

Urine cultures were retrieved from EMR and matched on day 0 to 5 from index visit. Urine cultures were analysed in 11,655 cases (55%). *E. coli* was the predominant bacterium detected in 3529 (30%) of the urine cultures. The second and third most common bacteria were *Klebsiella species* in 466 (4%) and *Enterococcus species* in 402 (3%), respectively. In 2066 (18%), other single pathogens were found, and in 805 (7%) of the samples, mixed growth was reported. No bacterial growth was reported in 4387 (38%) of cases. We were not able to retrieve data on antibiotic susceptibility.

Urine-dipsticks were positive for nitrite in 34% of analysed samples (analysed in 47% of index visits) and for leukocytes in 74% of samples (analysed in 54% of index visits) (Table 1).

Ciprofloxacin was the most used antibiotic, prescribed in 10,956 cases (52%), followed by pivmecillinam 5234 (25%), and nitrofurantoin 3223 (15%) (Table 1). For visits in hospital care, 70% of prescriptions were for ciprofloxacin, compared with 48% in PHC. Antibiotic treatment duration is presented in Table 1.

Therapy failure was registered in 192 (0.9%) and recurrence in 1277 (6%) of the cases (Table 1). Median age among men with therapy failure was 69 years IQR (59–74) and in recurrence 67 years IQR (58–73).

Complications within 30 days from index antibiotic prescribing were registered in 121 (0.6%) of cases (Table 1). Median age among these cases was 67 years IQR (58–72). Sixty-eight per cent of cases with complications were treated in hospital care. The most common complication was pyelonephritis, which was diagnosed in 99 cases (82% of the cases with complications). All 22 cases (18% of complications) of sepsis were diagnosed and treated in hospital care.

Therapy failure, recurrence and complications were more common in patients with growth of bacteria in urine as compared with patients with no growth of bacteria, therapy failure 2.67 (95% CI 1.74–4.14), recurrence 2.50 (95% CI 2.1–3.0) and complications 3.87 (95% CI 2.1–7.1). We did not find any association between therapy failure, recurrence and complications in relation to whether urine cultures had been taken or not.

Therapy failure and recurrence were more common in patients treated with narrow-spectrum antibiotic and trimethoprim (*p* < 0.001) (Table 2). There was no association between a raised CRP (≥20 mg/L) and therapy failure or recurrence but for complication there was an association, OR 2.8 (95% CI 1.9–4.1) (Table 3). Older age (>55 years) was associated with increased risk for therapy failure OR 1.6 (95% CI 1.2–2.3) recurrence OR 1.7 (95% CI 1.5–2.0) and complications OR 1.7 (95% CI 1.1–2.7) (Table 3).

**Table 2.** Therapy failure, recurrence and complications in relation to prescribed index antibiotic.

	Narrow-spectrum antibiotic^a^ *n* = 8457 *n* (%)	Broad-spectrum antibiotic^b^ <10 days *n* = 920 *n* (%)	*p*-value^c^	Broad- spectrum antibiotic^d^ ≥10 days *n* = 11 747 *n* (%)	*p* Value^e^
Therapy failure	103 (1.2)	11 (1.2)	0.92	78 (0.7)	<0.001
Recurrence	779 (9.2)	61 (6.6)	0.02	437 (3.7)	<0.001
Complication	45 (0.5)	4 (0.4)	0.89	72 (0.6)	0.48

*p-*values were calculated using the Chi-square test.

^a^ Pivmecillinam, nitrofurantoin.

^b^ Trimethoprim.

^c^ Comparison between narrow-spectrum antibiotic and broad-spectrum antibiotic <10 days (trimethoprim).

^d^ Ciprofloxacin, trimethoprim sulfamethoxazole.

^e^ Comparison between narrow-spectrum antibiotic and broad-spectrum antibiotic ≥10 days (ciprofloxacin, trimethoprim/sulfamethoxazole).

**Table 3.** Risk factors for therapy failure, recurrence and complications.

	Therapy failure *n* = 192		Recurrence *n* = 1277		Complication *n* = 121	
	Crude OR (95% CI)	Adjusted^a^ OR (95% CI)	Crude OR (95% CI)	Adjusted^a^ OR (95% CI)	Crude OR (95% CI)	Adjusted^a^ OR (95% CI)
Age over 55 years	1.6 (1.1–2.3)	1.6 (1.2–2.3)	1.6 (1.4–1.9)	1.7 (1.5–2.0)	1.9 (1.2–2.9)	1.7 (1.1–2.7)
CRP ≥20 mg/L	1.1 (0.8–1.5)	1.4 (1.0–1.9)	0.7 (0.6–0.9)	1.0 (0.9–1.2)	2.7 (1.9–3.9)	2.8 (1.9–4.1)
Broad-spectrum antibiotic ≥10 days	Ref	Ref	Ref	Ref	Ref	Ref
Broad-spectrum antibiotic <10 days	1.8 (1.0–3.4)	1.9 (1.0–3.7)	1.8 (1.4–2.4)	1.9 (1.4–2.5)	0.7 (0.3–1.9)	0.9 (0.3–2.5)
Narrow-spectrum antibiotic <10 days	1.8 (1.4–2.5)	2.0 (1.5–2.8)	2.6 (2.3–3.0)	2.7 (2.4–3.1)	0.9 (0.6–1.3)	1.2 (0.8–1.8)

CI: confidence interval; OR: odds ratio; CRP: C-reactive protein; Narrow-spectrum antibiotic treatment, pivmecillinam or nitrofurantoin; Broad-spectrum antibiotic treatment <10 days, trimethoprim; Broad-spectrum antibiotic treatment ≥ 10 days, trimethoprim/sulfamethoxazole or ciprofloxacin. Missing cases, CRP 12,345 (58.4%).

^a^ Method: Log regression enter, adjusted for age, CRP, narrow-spectrum antibiotic, broad-spectrum antibiotic <10 days and broad-spectrum antibiotic ≥10 days. Broad-spectrum antibiotic ≥10 days was used as reference.

There were no significant differences between the incidence of therapy failure, recurrence and complications between Skåne county and the four counties with complete data. The incidence of complications was low for all counties, in Skåne county 0.6% with both narrow- and broad-spectrum antibiotic treatment and for the four complete counties 0.5% with narrow-spectrum antibiotic and 0.6% with broad-spectrum antibiotic treatment.

## Discussion

### Statement of the principal findings

This study included 16,555 men, which were aged 18–79 years with lower UTI in PHC and hospital care, did not show any difference in complication rates between those who received narrow- and broad-spectrum antibiotic treatment. Patients prescribed narrow-spectrum antibiotics and trimethoprim had higher odds of having a reconsultation because of therapy failure and recurrence.

### Strengths and weaknesses of the study

This is the first study with the aim to compare treatment outcome between narrow-spectrum and broad-spectrum antibiotic treatment in men with lower UTI. It is also one of few studies on UTI in men that includes over 16,000 patients. Another strength is that we included all men that met the inclusion criteria for UTI in five different counties in Sweden during five consecutive years. We used defined criteria for inclusion, antibiotic prescribing therapy failure, recurrence and complications, which makes the results reproducible. We believe that, despite the lack of an RCT in this area, the findings presented in this study are useful evidence on this topic.

Few studies concerning antibiotic treatment and duration of treatment in men have been performed and there are few studies describing the incidence of complications. When planning this study, we estimated that the frequency of complications was 2% based on incidence of complications in women and former studies [13–15]. Our results show a much lower incidence of complications, which could, except for actual differences between men and women, be caused by differences in diagnoses included in the definition complications, different patient groups studied or factors that we are not aware of. The lack of data on index visits in hospital care with any subsequent therapy failure and recurrence in Skåne county could cause selection bias and the study could be under powered for therapy failure and recurrence. Therefore, we performed a sensitivity analysis and did not find any difference between outcome measures from Skåne county and the four counties with complete five-year data from PHC and hospital care.

A limitation with the study is the retrospective study design. We did not have information about clinical symptoms. Furthermore, we were unaware of how the clinical diagnosis was made, if the patient had a symptomatic UTI or if the UTI diagnosis instead represented a check-up consultation after a former UTI or asymptomatic bacteriuria (ABU). Urine cultures were only performed in 55% of cases. In order to minimise the risk of invalid lower UTI diagnosis, we only included registered index UTI visits with adjacent antibiotics within five days after consultation. We also excluded all cases that had an antibiotic prescription within 30 days prior to the index visit.

With age, men acquire structural and functional abnormalities of the urinary tract such as prostatic hyperplasia that can impair normal voiding and can cause UTI [16]. The engagement of the prostate gland in male UTI is a potential confounder, since it can be difficult to differentiate between a bacterial UTI or a prostatitis in clinical practice. This is an issue that is often discussed regarding diagnosis and choice of treatment in UTI in men and could cause indication bias.

We did not have data on potential risk factors for recurrence and complications such as instrumentation of the urinary tract and comorbidity. No propensity score (matching) was used. Patients that were prescribed ciprofloxacin could have been more affected or more fragile from the beginning (selection bias). We lack information about comorbidities, socioeconomic status, anamnestic factors and actual symptoms that would help to minimise these effects.

Data on susceptibility pattern in urine cultures were not possible to retrieve from the EMR. This is a limitation since knowledge about resistance patterns could affect antibiotic choice and outcome.

#### Findings in relation to other studies

In a retrospective study, Montelin et al. [17] assessed the clinical outcome from EMR in 129 men with lower UTI treated with nitrofurantoin or pivmecillinam, 45 men treated with trimethoprim were included for comparison. No significant difference in clinical outcome between the studied groups was found but recurrence within three months was more frequent with nitrofurantoin and pivmecillinam. No comparison with ciprofloxacin was done. In line with the above-mentioned study, our study with a larger population showed a significant difference in treatment outcome regarding recurrence (within 30 days) between the group treated with narrow- and broad-spectrum antibiotics.

No difference between UTI-related hospitalisation and death was seen in a retrospective cohort study with patients aged 65 years and older (27% were men) comparing nitrofurantoin with broad-spectrum antibiotics, although patients prescribed broad-spectrum antibiotics had lower odds of reconsultation and repeat prescription, which is in-line with our study [18]. In a cohort study, a higher risk for repeat prescription of antibiotics in male UTI after 4–28 days was seen in older men as compared to younger [19]. This concurs with our results where both reconsultation and recurrence were more common in older men and could be caused by anatomic changes in the male urinary tract.

In a large cohort study, which included 22,629 men with UTI aged 18–64 years, antibiotic repeat prescriptions were significantly less likely for UTI episodes treated with trimethoprim than other agents. This is in spite of a higher rate of resistance to trimethoprim in *E. coli* [19]. This difference was not seen in our study where therapy failure was 1.2% for all antibiotics except for ciprofloxacin with therapy failure in 0.6%.

According to the Swedish treatment guidelines for UTI in men, urine culture is always recommended. We find it surprising that cultures were only analysed in 55% of index visits. The reasons behind this are unclear. One of the explanations could be that collecting urine cultures is time consuming and since urine cultures are only collected for special indications in women they are sometimes forgotten in men. Another reason may be that the patients were less affected by the infection, which could make the physicians less prone to order urine cultures. To achieve better adherence to guidelines in men with lower UTI, empiric narrow-spectrum antibiotics should be prescribed, and a urine culture should always be performed. To follow up clinical cure and results from the urine culture, a follow-up contact with the patient should be scheduled. In case of therapy failure, the antibiotic choice should be reconsidered. The low rate of urine cultures is an important finding from the study and a vital issue to address in the future work towards better adherence to guidelines.

We did not find any association between therapy failure, recurrence and complications and analysed urine cultures. This phenomenon was also discussed in an article by Ingalsbe et al. [20]. Few urine cultures were also noted in a study from the United Kingdom where only 31% of men with a prescribed antibiotic had a recorded urine test [19].

The recommended treatment duration for the first-line antibiotics in lower UTI in men (pivmecillinam or nitrofurantoin) is seven days. In our study, median treatment duration for nitrofurantoin was five days, and for pivmecillinam, it was seven days. This indicates that treatment duration differs from treatment guidelines. The minimum duration of antibiotic treatment for lower UTI in men has not yet been determined [3] but a recent Danish retrospective study showed no significant difference in treatment failure between five- or seven-day regimens with pivmecillinam in men [21]. In a review article, the researchers could only identify one RCT [22] and one observational study [23] since the year 2000 addressing male UTI [24]. Concluding these studies, duration of therapy for acute UTI in men should be limited to seven to 14 days [24].

## Conclusions

Complications within 30 days after a clinical diagnosis of lower UTI in men 18–79 years were rare and there was no difference in incidence of complications among those treated with narrow- and broad-spectrum antibiotics. However, therapy failure and recurrence were more common among patients treated with narrow-spectrum antibiotics and trimethoprim. In spite of higher incidence of therapy failure and recurrence in the narrow-spectrum group, narrow-spectrum antibiotics seems to be a proper first choice antibiotic in male UTI patients, since there is no difference in serious complications such as pyelonephritis and sepsis. Considering the increased rates of drug-related adverse events and the selection of drug-resistant organisms following broad-spectrum antibiotic use, these antibiotics should be preserved for serious infections.

Future studies are needed to evaluate the most appropriate antibiotic treatment and duration of therapy for UTI in men.

## Supplementary Material

Supplemental Material
