# Supplementary material for: Different antibiotic regimes in men diagnosed with lower urinary tract infection – a retrospective register-based study
Source: Scand J Prim Health Care. 2020 Jul 20;38(3):291–9. doi: 10.1080/02813432.2020.1794409 (PMC7470089; doi:10.1080/02813432.2020.1794409)
Supplement: Supplemental Material [file IPRI_A_1794409_SM4212.doc]

# **Supplementary material**

**Included Urinary tract infection diagnosis codes**

| **Acute cystitis** | |
| --- | --- |
| *Code* | *Description* |
| N30.-P | Cystitis  Urinary tract infection |
| N30.0 | Acute cystitis |
| N30.8 | Other cystitis |
| N30.9 | Cystitis, unspecified |
| N39.0 | Urinary tract infection, site not specified |
| N39.0X | Urinary tract infection UNS |

**Included diagnosis of complications to UTI**

| **Acute pyelonephritis** | |
| --- | --- |
| *Code* | *Description* |
| N12.-P | Pyelonephritis |
| N12.9 | Tubulo-interstitial nephritis, not specified as acute or chronic |
| N10.9 | Acute Tubulo-interstitial nephritis |
| A41-P | Sepsis |
| A419 | Sepsis, unspecified |
| A418 | Other specified sepsis |
| A415 | Sepsis due to other Gram-negative organisms |
